# Incidence, Clinical Spectrum, Risk Factors and Impact of HIV-Associated Immune Reconstitution Inflammatory Syndrome in South Africa

**DOI:** 10.1371/journal.pone.0040623

**Published:** 2012-11-12

**Authors:** Lewis John Haddow, Mahomed-Yunus Suleman Moosa, Anisa Mosam, Pravi Moodley, Raveen Parboosing, Philippa Jane Easterbrook

**Affiliations:** 1 Centre for Sexual Health and HIV Research, Research Department of Infection and Population Health, University College London, London, United Kingdom; 2 Department of Infectious Diseases, Nelson R. Mandela School of Medicine, University of KwaZulu-Natal, Durban, South Africa; 3 Department of Dermatology, Nelson R. Mandela School of Medicine, University of KwaZulu-Natal, Durban, South Africa; 4 Department of Virology, Nelson R. Mandela School of Medicine, University of KwaZulu-Natal, Durban, South Africa; 5 National Health Laboratory Services, Inkosi Albert Luthuli Central Hospital, Durban, South Africa; South Texas Veterans Health Care System and University Health Science Center San Antonio, United States of America

## Abstract

**Background:**

Immune reconstitution inflammatory syndrome (IRIS) is a widely recognised complication of antiretroviral therapy (ART), but there are still limited data from resource-limited settings. Our objective was to characterize the incidence, clinical spectrum, risk factors and contribution to mortality of IRIS in two urban ART clinics in South Africa.

**Methods and Findings:**

498 adults initiating ART in Durban, South Africa were followed prospectively for 24 weeks. IRIS diagnosis was based on consensus expert opinion, and classified by mode of presentation (paradoxical worsening of known opportunistic infection [OI] or unmasking of subclinical disease). 114 patients (22.9%) developed IRIS (36% paradoxical, 64% unmasking). Mucocutaneous conditions accounted for 68% of IRIS events, mainly folliculitis, warts, genital ulcers and herpes zoster. Tuberculosis (TB) accounted for 25% of IRIS events. 18/135 (13.3%) patients with major pre-ART OIs (e.g. TB, cryptococcosis) developed paradoxical IRIS related to the same OI. Risk factors for this type of IRIS were baseline viral load >5.5 vs. <4.5 log_10_ (adjusted hazard ratio 7.23; 95% confidence interval 1.35–38.76) and ≤30 vs. >30 days of OI treatment prior to ART (2.66; 1.16–6.09). Unmasking IRIS related to major OIs occurred in 25/498 patients (5.0%), and risk factors for this type of IRIS were baseline C-reactive protein ≥25 vs. <25 mg/L (2.77; 1.31–5.85), haemoglobin <10 vs. >12 g/dL (3.36; 1.32–8.52), ≥10% vs. <10% weight loss prior to ART (2.31; 1.05–5.11) and mediastinal lymphadenopathy on pre-ART chest x-ray (9.15; 4.10–20.42). IRIS accounted for 6/25 (24%) deaths, 13/65 (20%) hospitalizations and 10/35 (29%) ART interruptions or discontinuations.

**Conclusion:**

IRIS occurred in almost one quarter of patients initiating ART, and accounted for one quarter of deaths in the first 6 months. Priority strategies to reduce IRIS-associated morbidity and mortality in ART programmes include earlier ART initiation before onset of advanced immunodeficiency, improved pre-ART screening for TB and cryptococcal infection, optimization of OI therapy prior to ART initiation, more intensive clinical monitoring in initial weeks of ART, and education of health care workers and patients about IRIS.

## Introduction

Immune reconstitution inflammatory syndrome (IRIS) or immune reconstitution disease is a widely recognised phenomenon that occurs in 10−27% of patients initiating antiretroviral therapy (ART) [1], [2], [3], [4], [5], [6], [7], [8]. The syndrome may present in two different ways: “paradoxical” IRIS, when an opportunistic infection (OI) diagnosed pre-ART initially responds to treatment but then deteriorates following ART initiation; or “unmasking” IRIS, where disease that is not clinically apparent prior to ART is triggered by ART initiation, often with unusual or florid inflammatory features [1], [9] in association with rapid restoration of pathogen-specific immune responses [10]. The clinical spectrum is extremely diverse, and IRIS has been reported for at least 25 different infections, 2 tumours and 18 other non-infectious conditions [11]. Pooled incidence rates of paradoxical IRIS are 17% for tuberculosis (TB) (range 8–45%) [7], [12], [13], [14], [15], [16], [17], [18], 20% for cryptococcosis (range 8–49%) [7], [19], 17% for progressive multifocal leukoencephalopathy [20], 7–31% for Kaposi's sarcoma (KS) [21], [22], and 12% for herpes zoster [23]. Data on the incidence of unmasking IRIS are limited, but rates of 1–5% for TB IRIS [1], [24], [25] and 1–2% for cryptococcal IRIS [19] have been reported from Uganda and South Africa.

Most studies have examined risk factors for paradoxical and unmasking IRIS as one entity [1], [2], [3], [5], [6], [26], [27], or for paradoxical IRIS specific to TB [13], [15], [16], [17], [18], [28], [29], cryptococcosis [30], [31], [32] or KS [22]. *Patient*- and *ART-related* risk factors include lower baseline absolute and percentage CD4^+^ cell count [1], [2], [3], [6], [16], [18], [26], [31], lower baseline haemoglobin or haematocrit [5], [22], [27], greater magnitude of CD4^+^ count increase [6], [15], [32], [33] or viral load (VL) reduction [3], [13], [15], [22], [26], [28], [30], [33] on ART, and use of a boosted protease inhibitor [26]. *Pathogen-related* factors include shorter duration of anti-tuberculous or anti-cryptococcal therapy prior to ART initiation [13], [16], [28], [29], [30], [31], extrapulmonary or disseminated TB [15], [17], [18], [29], higher fungal burden in cryptococcosis [30], [32], higher plasma KS-associated herpes virus (KSHV) load [22], and higher number of prior OIs [27].

IRIS may be a greater problem in resource-limited settings (RLS), where there is large scale rollout of ART in populations with high rates of advanced immunodeficiency and co-morbidity. There are still limited data on the epidemiology of IRIS from RLS, and debate as to its programmatic importance, particularly its contribution to the high early mortality seen in the first year of ART.

Our objective was to document comprehensively the epidemiology of IRIS, in a large prospective cohort of patients initiating ART in two typical urban clinics in South Africa, including incidence, clinical spectrum, risk factors, clinical outcome, and contribution to hospitalisation and death. We postulated that risk factors would differ according to IRIS subtype, in particular that advanced immunodeficiency and duration of OI therapy prior to ART (as indirect measures of pathogen burden) would be risk factors for paradoxical OI IRIS, while clinical and laboratory features consistent with undiagnosed OIs would be risk factors for unmasking OI IRIS.

## Methods

### Ethics statement

The study was approved by the University of KwaZulu-Natal Biomedical Research Ethics Committee in November 2006, ref. E024/06. All patients were provided with a written and verbal explanation of the study by bilingual research staff in English and/or isiZulu (the predominant first language in KwaZulu-Natal), and gave written informed consent.

### Study participants

Adult (≥18 years) HIV-1-infected ART naïve patients due to initiate ART according to 2004 South African national guidelines (CD4^+^ count of less than 200 cells/µL or WHO stage 4 disease [34]) were invited to participate in a prospective observational cohort study at two clinics (King Edward VIII and RK Khan Hospitals) in Durban, South Africa between December 2006 and October 2007. King Edward VIII Hospital serves mainly an urban population, while RK Khan Hospital serves a more mixed urban and rural population with of poorer health and socioeconomic status. The same protocols were applied at both sites. ART regimens were stavudine and lamivudine with efavirenz or nevirapine, given with co-trimoxazole prophylaxis (either one month before or at ART initiation) and multivitamins.

### Baseline assessments

All patients underwent pre-ART assessment by English- and isiZulu-speaking research nurses. Data collected using a standardised questionnaire included detailed current and recent symptom history (elicited by direct questioning), past medical history, and brief physical examination. Baseline laboratory investigations were CD4^+^ and CD8^+^ counts (Flowcare™ PLG CD-4 Assay, Beckman Coulter, Ireland), HIV-1 RNA viral load (VL) (Nuclisens® EASYQ HIV-1 V1.1 Assay, Biomerièux, Lyon, France), full blood count, renal function (creatinine), liver enzymes (aspartate and alanine transaminase, and bilirubin), albumin, glucose, hepatitis B surface antigen (AXSYM system v.2, Abbott Diagnostics, Weisbaden, Germany) and syphilis serology (Rapid Plasma Reagin with confirmation by *Treponema pallidum* haemagglutination assay). Additional baseline serum and plasma samples were tested retrospectively at the end of study follow-up for hepatitis C IgG (ADVIA Centaur, Bayer Diagnostics, New York, USA), herpes simplex type 2 IgG (HerpeSelect 2 ELISA, Focus Diagnostics), cryptococcal antigen (CrAg) (semi-quantitative latex agglutination assay; agglutination titres ≥1∶4 were considered positive), and C-reactive protein (DXC Beckman). Symptomatic patients were evaluated further according to clinician judgement and managed in accordance with South African national guidelines [34]. Those with suspected TB were screened with chest x-ray and sputum examination for acid-fast bacilli, and if positive received a median of 12 weeks of anti-tuberculous therapy prior to ART initiation. Cryptococcosis was treated with 2 weeks of intravenous amphotericin B or high-dose fluconazole, followed by fluconazole consolidation and maintenance therapy. KS-specific chemotherapy was not widely available in Durban in 2007.

### Follow-up assessment and classification of clinical events

Participants were reviewed after 2, 4, 8, 12, 16, 20 and 24 weeks of ART using standardised questionnaires, with additional medical assessments as required, and telephone calls to ascertain events in patients with missed visits. All clinical events, including episodes of new or worsening symptoms, hospital admissions, deaths and changes in ART regimen were recorded. CD4^+^ and CD8^+^ counts, VL, full blood count, liver enzymes, albumin and renal function were measured at the time of each event, and routinely at 12 and 24 weeks.

All patients with clinical events were evaluated by at least one of the investigators in person (usually LH), other than in a few cases where information was obtained from other medical or nursing staff, medical chart review, or patients' relatives. All events were then reviewed by ≥2 investigators (LH, YM, PE, AM), and the diagnosis of IRIS established through consensus review, after consideration of all available clinical and laboratory data. Indeterminate cases were discussed between all three investigators and/or a dermatologist (AM).

Events were then categorised into a number of groups: IRIS (either probable IRIS, where IRIS was the most likely of possible causes, or possible IRIS, where IRIS was plausible but competing explanations were equal or stronger in likelihood, or the diagnosis could not be resolved with available information) or non-IRIS (includes new infection; drug toxicity; progression of pre-ART disease; other conditions). We subsequently combined Possible and non-IRIS into a single non-IRIS category, as the preponderance of evidence suggested that IRIS was unlikely in these cases.

A detailed description of follow-up procedures and criteria used for classification of suspected IRIS has been reported previously [24], [35]. In brief, diagnostic criteria for probable IRIS included relapse, exacerbation or new onset of an infectious or inflammatory condition in association with evidence of at least partial immune recovery, not readily explained by another cause. Additional features that provided support for IRIS included: temporal relationship with ART initiation (symptom onset <3 months into ART); resolution without change in OI therapy; clinical course not consistent with pre-ART disease; no recent cessation of therapy for a pre-existing condition; plausibility that the antigen or pathogen was present prior to ART initiation; unusual or florid presentation; and exclusion of other explanations for the deterioration such as drug toxicity and drug-resistant organisms.

Initial categorisation of events as IRIS or non-IRIS was done without prior knowledge of ART response. When CD4 and viral load data were subsequently available at the end of the study, we considered that absence of any rise in the CD4 cell count (±10 cells) at clinical event or of a decline in the viral load (±0.25 log) made a diagnosis of IRIS unlikely, and such events were therefore categorised as non-IRIS.

IRIS cases were classified according to mode of presentation (paradoxical or unmasking) and underlying diagnosis (major OIs – TB, WHO stage 4 conditions and viral hepatitis – or mucocutaneous if they affected primarily the skin, oropharynx or lower genital tract). Therefore, the four subtypes of IRIS were: paradoxical IRIS of major OIs (Paradox-OI), unmasking of major OIs (Unmask-OI), paradoxical mucocutaneous IRIS (Paradox-MC), and unmasking mucocutaneous IRIS (Unmask-MC). Possible and non-IRIS cases were categorised by underlying cause: new infection; drug toxicity; progression of pre-ART disease; other conditions. For recurrent condition such as genital herpes, we used additional criteria to differentiate paradoxical IRIS from non-IRIS recurrent disease: increased frequency and severity of recurrences, and where relevant poor response to therapy, compared to pre-ART.

### Statistical analysis

Incidence rate and risk, time to event, and outcomes (death, hospital admission, ART switch or interruption) were calculated for each of the four IRIS subtypes and non-IRIS clinical events. Patients at risk of paradoxical IRIS were those with a clinically apparent OI or skin condition just prior to ART initiation (regardless of prior serological evidence of infection). Patients at risk of unmasking IRIS for a specific condition were those without evidence of clinically active disease prior to ART. The denominator for unmasking IRIS related to infections such as HSV-2 and cryptococcosis was not restricted to those who were seropositive. In this way, incidence calculations could be compared with other studies and across all conditions. For non-IRIS clinical events, the denominator was all patients in the cohort. Cox proportional hazards models were used to identify risk factors for each of the four IRIS subtypes, and hazard ratios (HR) were estimated for each variable in univariate analyses, followed by multivariable analyses of variables with *P*<0.10 in univariate analyses. All adjusted HR (aHR), two-sided 95% confidence intervals (CI) were calculated and graphs were plotted using STATA® software version 10.

There were 4 groups of risk factors for development of IRIS (all baseline unless stated): *Patient related* (e.g. age, sex, body mass index, full blood count, renal and liver function); *HIV-related* (e.g. WHO stage [not Paradox-OI], absolute and percentage baseline CD4^+^ and CD8^+^ counts, and log VL); *ART-response associated* (e.g. ART drug regimen, CD4^+^ and VL response at 12 and 24 weeks); and *pathogen/disease-related* (e.g. clinical and laboratory measures of disease burden, such as CrAg titre, [clinical site and extent of disease or quantitative measure; Paradox-OI only], extent of disease on chest x-ray [Paradox-OI only], duration of prior OI anti-infective therapy or prophylaxis prior to ART [Paradox-OI only], C-reactive protein, number of previous OIs, current or previous TB [MC-IRIS only], and systemic symptoms [cough, weight loss, night sweats, fever], mucocutaneous symptoms [extent, duration and severity of skin and orogenital lesions], cryptococcal antigen (CrAg) positivity [OI IRIS only], and use of co-trimoxazole and fluconazole prophylaxis prior to ART).

## Results

### Patient characteristics (Table 1)

Of 498 participants, at ART initiation 375 (75.3%) were female, median age 35 years (interquartile range [IQR] 30–41 years), median CD4^+^ count 106 cells/µL (53–165 cells/µL), median VL 5.0 log_10_ copies/mL (4.4–5.6 log_10_ copies/mL), 344 (69.1%) WHO stage 3 or 4 disease, and 102 (20.5%) known TB co-infection. 243 (48.8%) had a positive TB symptom score [36], of whom 189 (77.8%) screened negative for active TB. Eight (1.6%) had recent cryptococcosis, 7 (1.4%) had KS, 39 (7.8%) were hepatitis B surface antigen positive, and 154 (30.9%) had a history of genital ulcer disease. During 214 patient-years (PY) of follow-up, 25 (5.0%) died at a median 83 days after ART initiation (IQR 28–107 days), and 21 (4.2%) were lost to follow-up. At 24 weeks, the median CD4^+^ cell count had risen to 193 cells/μL (IQR 135–270 cells/μL) and 343/423 (81.1%) patients remaining under follow-up had VL <50 copies/mL.

**Table 1:** Demographic, clinical and laboratory characteristics of 498 study participants at ART initiation.

Characteristic		Median	Inter-quartile range	Number	%
Age, years		34.5	29.4–40.5		
Female				375	75.3
Body mass index (kg/m^2^)		23.2	20.5–27.1		
WHO stage	1			67	13.5
	2			88	17.7
	3			280	56.2
	4			63	12.7
**OI history (in past year unless stated)**	Lifetime history of TB			220	44.2
	Current TB, on anti-TB therapy			102	20.5
	Duration of anti-TB therapy prior to ART (days)	81	56–152		
	Cryptococcosis *			8	1.6
	Duration of current cryptococcosis therapy (days)	171	92–248		
	Kaposi's sarcoma **			7	1.4
	Herpes zoster (last 5 years)			126	25.3
	Genital ulcers			155	31.1
	Any skin complaint			275	55.2
	Folliculitis			142	28.5
**Positive serology**	Hepatitis B surface antigen			39	7.8
	Hepatitis C IgG			1	0.2
	Syphilis RPR/TPHA			14	2.8
	Cryptococcal antigen ***			41	8.2
**Symptoms (at least 2 weeks)**	Weight loss >10%			188	37.7
	Night sweats			164	32.9
	Fever			134	26.9
	Cough			191	38.4
	Positive tuberculosis symptom score ****			243	48.8
	Unexplained diarrhoea ≥4 weeks			104	20.9
**ART regimen**	Stavudine, lamivudine, and efavirenz			312	62.7
	Stavudine, lamivudine, and nevirapine			186	37.3
**Laboratory**	Haemoglobin (g/dL)	11.2	10.0–12.4		
	CD4^+^ count (cells/µL)	106	53–165		
	CD4^+^%	9	5–13		
	CD8^+^ count (cells/µL)	666	447–1039		
	CD8^+^%	63	54–72		
	Viral load (log_10_ copies/mL)	4.98	4.38–5.56		

* All 8 cryptococcosis patients were receiving oral fluconazole maintenance treatment at ART initiation following induction therapy with amphotericin B (*n* = 5) or high-dose fluconazole (*n* = 3).

** Sites of Kaposi's sarcoma (KS): oral (*n* = 4), cutaneous (*n* = 2), or both (*n* = 1). Only one patient had received KS-specific chemotherapy.

*** 8 (19.5%) of 41 serum CrAg positive patients identified from retrospective testing of baseline samples had a prior history of cryptococcal disease. The remainder were asymptomatic at baseline, of whom 2 (6.1%) developed unmasking cryptococcal IRIS.

**** Defined as the presence ≥2 of the following symptoms for ≥2 weeks: cough, fever, night sweats, weight loss.

ART: anti-retroviral therapy; CrAg: cryptococcal antigen; RPR: rapid plasma reagin; TB: tuberculosis; TPHA: *Treponema pallidum* haemagglutination assay.

### Incidence, clinical spectrum and timing of IRIS

Over the 24 weeks of follow-up, there were 620 clinical events, of which 139 (22.4%) in 114 patients were considered probable IRIS (cumulative incidence 22.9%; rate 64.8/100 PY [Table 2]). An additional 111 (17.9%) initially considered possible IRIS were subsequently considered non-IRIS as the preponderance of evidence suggested IRIS was unlikely. Five events originally classified as probable IRIS were excluded because of the absence of any virological or immunological response at the time of the event. Non-IRIS events accounted for the majority of post-ART events and comprised 161 new infections (incidence 26.7%), 144 cases of pre-existing disease with relapse, progression or persistence of symptoms (22.5%), 75 drug toxicities (14.1%), and 101 other problems (17.7%).

**Table 2:** Incidence of IRIS and non-IRIS clinical events, and IRIS subtypes over the 24 weeks after ART initiation (*n* = 498 patients, 620 events).

Event		No. events	Patients with event/Patients at risk	% incidence (95% CI)	Onset of event, median days after starting ART (IQR)
Any IRIS		139	114/498	22.9 (19.3–26.8)	15 (7–48)
New infection *		161	133/498	26.7 (22.9–30.8)	52 (21–91)
Pre-existing disease (Relapse, progression or persistent symptoms)		144	112/498	22.5 (18.8–26.2)	16 (7–42)
Drug toxicity		75	70/498	14.1 (11.1–17.4)	25 (7–69)
Other **		101	88/498	17.7 (14.3–21.0)	24 (7–62)
*Paradoxical OI IRIS*	All major OIs	19	18/135	13.3 (8.1–20.3)	10 (7–21)
	Tuberculosis	15	14/102	13.7 (7.7–22.0)	10 (7–21)
	Cryptococcosis	2	2/8	25.0 (3.2–65.1)	
	Kaposi's sarcoma	1	1/7	14.3 (0.4–57.9)	
	Oesophagitis	1	1/21	4.8 (0.1–23.8)	
*Unmasking OI IRIS*	All major OIs	25	25/498	5.0 (3.3–7.3)	12 (7–46)
	Tuberculosis	19	19/396	4.8 (2.9–7.4)	12 (7–49)
	Cryptococcosis	2	2/490	0.4 (0.05–1.5)	
	Kaposi's sarcoma	0	0/491	0	
	Other major OI ***	4	4/498	0.8 (0.2–2.0)	
*Paradoxical mucocutaneous IRIS*	All mucocutaneous conditions	31	30/322	9.3 (6.4–13.0)	14 (7–28)
	Folliculitis	12	12/142	8.5 (4.4–14.3)	7 (7–21)
	Warts	3	3/38	7.9 (1.7–21.4)	
	Genital herpes simplex	5	5/155	3.2 (1.1–7.4)	
	Herpes zoster	3	3/126	2.4 (0.5–6.8)	
*Unmasking mucocutaneou IRIS*	All mucocutaneous conditions	64	52/498	10.4 (7.9–13.5)	23 (7–53)
	Folliculitis	26	26/356	7.3 (4.8–10.5)	38 (9–58)
	Warts	7	7/460	1.5 (0.6–3.1)	
	Genital herpes simplex	8	8/343	2.3 (1.0–4.5)	
	Herpes zoster	6	6/372	1.6 (0.6–3.5)	

The denominators for paradoxical IRIS relating to each opportunistic condition were defined by the number of patients with clinically apparent disease at or just prior to ART initiation. In contrast, patients at risk of unmasking IRIS were those without clinically apparent disease prior to ART. For non-IRIS clinical events, the denominator was the total number of patients in the cohort. (*n* = 498). ART: anti-retroviral therapy; CI: confidence interval; IQR: interquartile range; IRF: immune reconstitution folliculitis; IRIS: immune reconstitution inflammatory syndrome; OI: opportunistic infection; PY: patient-years.

* Most common new non-IRIS infections were: upper or lower respiratory tract infection (*n* = 53); skin infections (fungal [*n* = 14], bacterial [*n* = 8] or other [*n* = 8]); enteric infection (*n* = 21); vaginal infection (*n* = 20); other genitourinary infections (*n* = 12); sepsis/bacteraemia (*n* = 5).

** Most common “other” non-IRIS events were: skin conditions (*n* = 21); non-specific constitutional symptoms and headache (*n* = 19); arthropathy/musculoskeletal problems (*n* = 16); genitourinary conditions (*n* = 14); gastrointestinal complaints (*n* = 10); trauma (*n* = 6). For arthropathy, alternative explanations included: trauma; arthralgia associated with a non-specific viral infection; pyrazinamide toxicity; myalgia and arthralgia without any signs or apparent aetiology.

*** Includes one case each of oesophagitis, strongyloidiasis, sarcoidosis and non-tuberculous mycobacterial infection.

**Figure 1:**
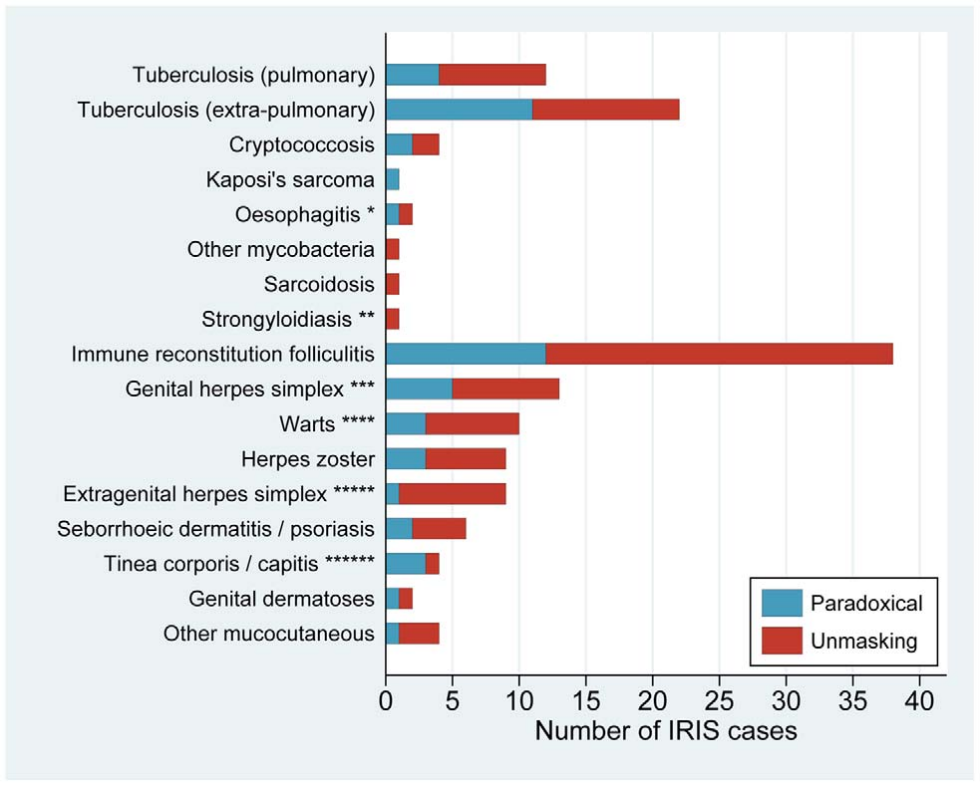
Clinical spectrum of 139 IRIS events by mode of presentation (paradoxical or unmasking) and underlying diagnosis. * Features of oesophagitis IRIS were odynophagia with significant anorexia, endoscopic findings, or haematemesis, with a clinical course consistent with paradoxical or unmasking IRIS and not typical of reflux or other common causes. ** The strongyloides IRIS case was unusual in its severity (it was diagnosed post mortem); the arguments that this case was IRIS have been published in a case report [67]. *** Based on clinical evidence of genital ulcer disease (GUD), pre-ART serologic evidence of herpes simplex virus (HSV)-2 infection, and exclusion of syphilis or confirmation of HSV-2 by polymerase chain reaction on ulcer swab. A diagnosis of unmasking HSV-IRIS was based on new onset GUD despite pre-ART serologic evidence of herpes simplex virus (HSV)-2 infection. A diagnosis of paradoxical HSV-IRIS was based on increasing frequency and/or severity of episodes of known recurrent genital herpes, relative to pre-ART. Anti-herpetic therapy was not available in this setting. **** Genital, orolabial or generalised warts. Unmasking IRIS involving genital warts was supported by a history of sexual abstinence since prior to ART initiation. ***** Intra-oral pain and ulceration (n = 6), or extra-oral ulceration (n = 3, one involving most of the face), with virological confirmation of HSV-1 by polymerase chain reaction, or no other likely causative organism. ****** Features suggestive of tinea IRIS were: unusually rapid spread of lesions, or marked inflammation. There were 18 more “typical” non-IRIS tinea cases that occurred during an interruption in ART, or where clinical history was insufficient to support an IRIS diagnosis.

Figure 1 shows the clinical spectrum of IRIS according to mode of presentation and underlying diagnosis. Overall, 89/139 IRIS events (64.0%) were unmasking in presentation, and 50 (36.0%) were paradoxical; 44 were related to major OIs (31.7%) and 95 (68.3%) were mucocutaneous. The commonest major OI associated with IRIS was TB (24.5% of all IRIS events; 15 paradoxical and 19 unmasking), followed by cryptococcosis (2.9%; 2 paradoxical and 2 unmasking). Immune reconstitution folliculitis (IRF) was the most frequent IRIS event overall (27.3%; 12 paradoxical and 26 unmasking), usually presenting as a pruritic, papular or pustular eruption with a central distribution. Viral mucocutaneous IRIS was also common (29.5%; 12 paradoxical and 29 unmasking), and included genital herpes, herpes zoster, extragenital herpes simplex, and warts.

Table 2 shows the incidence rate of different subgroups of IRIS. Of 135 patients with a pre-existing OI, 18 (13.3%) developed Paradox-OI IRIS, and the incidence was similar for TB (13.7%), cryptococcosis (25.0%) and KS (14.3%), although with wide confidence intervals for the latter two conditions. Of 322 patients with a history of mucocutaneous disease at baseline, 30 (9.3%) developed Paradox-MC IRIS, most frequently immune reconstitution folliculitis (IRF) (8.5%), warts (7.9%), genital herpes simplex (3.2%) and herpes zoster (2.4%). Out of 498 patients, 25 (5.0%) developed Unmask-OI IRIS and 52 (10.4%) developed Unmask-MC IRIS. The most common Unmask-OI IRIS events were TB (incidence 4.8%), followed by cryptococcosis (0.4%), and the most common Unmask-MC IRIS events were IRF (7.3%), genital herpes simplex (2.3%) and warts (1.5%).

**Figure 2:**
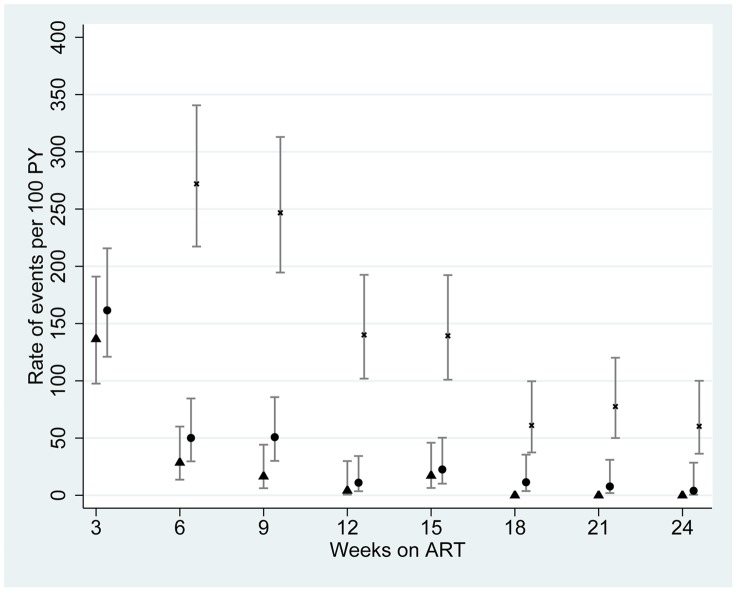
Rate of IRIS and non-IRIS events over 24 weeks from anti-retroviral therapy (ART). Rates are calculated per 100 patient-years (PY) in 3-week intervals, with 95% confidence intervals. Separate plots are shown for paradoxical IRIS (▴), unmasking IRIS (•) and non-IRIS events (×). Rate of non-IRIS events in the first 3 weeks was 741 events/100 PY (95% confidence interval 648–848) (data not plotted).

Figure 2 shows the rapidly declining incidence rates of paradoxical IRIS, unmasking IRIS, and non-IRIS clinical events over 24 weeks after ART initiation. 99 (71.2%) and 123 (88.5%) IRIS events occurred within the first 6 and 12 weeks after ART initiation respectively, compared with 59.0% and 80.9% for non-IRIS events. In the first 6 weeks, 25.5% of all events were due to IRIS, compared to only 8% of events in weeks 19–24. The median time to onset of IRIS after starting ART was 15 days (IQR 7–48 days), compared to 27 days (IQR 7–66 days) for non-IRIS events (p = 0.0048, Mann-Whitney test). Unmasking IRIS occurred slightly later (median 21 days, IQR 7–52 days) than paradoxical IRIS (11 days, IQR 7–24 days) (*P* = 0.043).

### Outcomes and impact of IRIS

**Figure 3:**
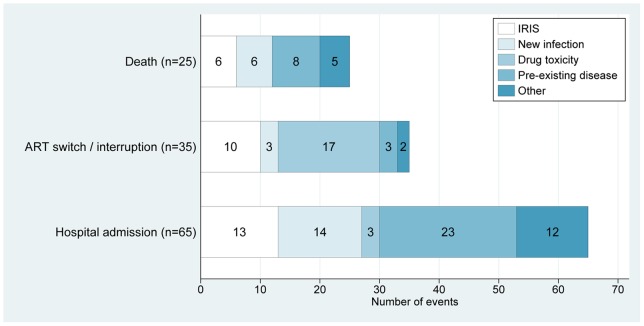
Major adverse outcomes within 24 weeks of starting antiretroviral therapy, with underlying cause.

Figure 3 shows the proportions of deaths, hospitalizations, ART switches and ART interruptions that were attributed to IRIS, compared to non-IRIS events. Non-IRIS events due to progression of pre-existing disease accounted for 23/65 (35%) hospital admissions and 8/25 (32%) deaths. Drug toxicity was the most common cause of ART switches (16/28; 57%) and interruptions (6/13; 46%).

Of the 25 deaths, six (24%) were caused by IRIS (unmasking of disseminated TB [n = 3], unmasking strongyloidiasis [n = 1], paradoxical TB/cryptococcosis [n = 1] and paradoxical KS [n = 1]). The 19 non-IRIS causes of death were: new infections (pneumonia [n = 3], bacterial meningitis [n = 1], other septicaemia [n = 2]); progression of pre-existing diseases (TB in a non-adherent patient [n = 1], carcinoma of the cervix [n = 1], chronic diarrhoea [n = 2], progressive cryptococcosis [n = 1], progressive demise of unknown cause [n = 1]); and other causes (pulmonary embolus [n = 2], suicide [n = 1], road traffic accident [n = 1], sudden death of unknown cause at home [n = 3] of whom one was not adherent to ART). Of the 65 hospital admissions, 13 (20%) were due to IRIS events: unmasking TB-IRIS (n = 7), paradoxical TB-IRIS (n = 2), cryptococcosis (n = 1), KS (n = 1), strongyloidiasis (n = 1), and severe genital warts requiring surgery (n = 1). Of the 43 patients with IRIS related to major OIs, 16.3% died (compared to 7.0% of those with any IRIS type, and 7.0% with a non-IRIS event) and 34.9% were hospitalised.

Thirteen (11.4%) patients with IRIS changed ART regimens, although this was not significantly different from those without IRIS (6.4%, *P* = 0.11). All IRIS-related changes in ART were due to initiation of rifampicin as part of TB therapy, requiring a switch from nevirapine to efavirenz. IRIS had no apparent impact on adherence to ART, with a similar proportion of patients with or without IRIS having >95% adherence based on pill counts at every study visit.

### Risk factors for IRIS

**Figure 4:**
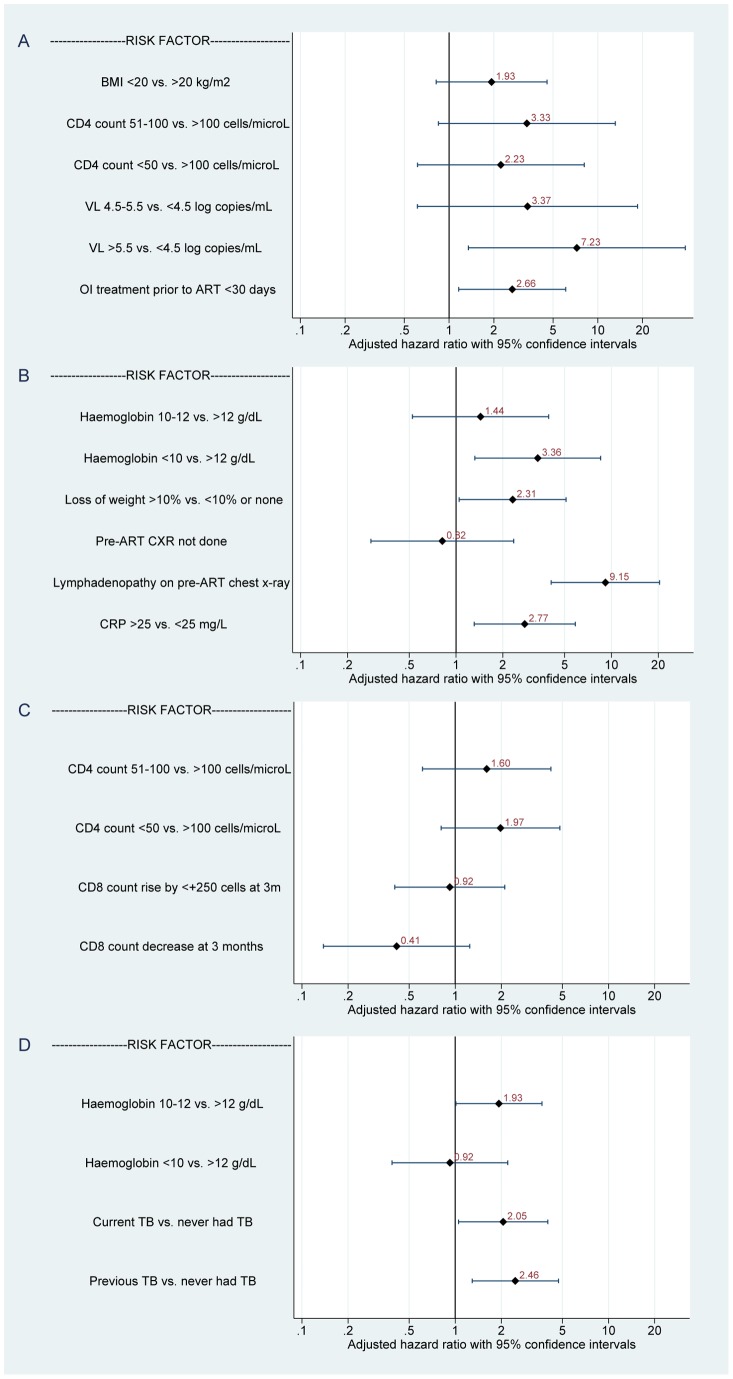
Multivariable analysis of risk factors for IRIS according to subtype. A, paradoxical IRIS related to major OIs (19 events in 135 patients [*n* = 15 TB]); B, unmasking IRIS related to major OIs (25 events in 498 patients [*n* = 19 TB]); C, paradoxical mucocutaneous IRIS (31 events in 418 patients); D, unmasking mucocutaneous IRIS (64 events in 498 patients). All variables were pre-ART unless stated. Potential risk factors were excluded from multivariable models if *P*≥0.10 on univariate analysis. LN: lymphadenopathy; OI: opportunistic infection.

In multivariable analysis, statistically significant risk factors for Paradox-OI IRIS (Figure 4a) were baseline VL >5.5 vs. <4.5 log_10_ copies/mL (aHR 7.23, 95% CI 1.35–38.76, *P* = 0.021) and ≤30 vs. >30 days of OI therapy (mainly TB) prior to ART (aHR 2.66, CI 1.16–6.09, *P* = 0.021). When analysed as a continuous variable, for each 1 log_10_ copies/mL increase in VL there was a 2.48-fold increased hazard (CI 1.43–4.31, *P* = 0.001). Additional risk factors of borderline statistical significance in univariate but not multivariable analyses were BMI <20 vs. ≥20 kg/m^2^ (crude HR 2.32, CI 0.94–5.70, *P* = 0.068; aHR 1.93, CI 0.82–4.56, *P* = 0.13), pre-ART CD4^+^ count ≤50 vs. >100 cells/µL (crude HR 3.51, CI 0.95–12.96, *P* = 0.060; aHR 2.23, CI 0.61–8.10, *P* = 0.22), and pre-ART CD4^+^ count 51–100 vs. >100 cells/µL (crude HR 3.75, CI 0.97–14.50, *P* = 0.056; aHR 3.33, CI 0.85–13.10, *P* = 0.085).

Risk factors for Unmask-OI IRIS on multivariable analysis (Figure 4b) were evidence of lymphadenopathy vs. no lymphadenopathy on pre-ART chest x-ray (aHR 9.15, CI 4.10–20.42, *P*<0.001), haemoglobin <10 vs. >12 g/dL (aHR 3.36, CI 1.32–8.52, *P* = 0.011); CRP ≥25 vs. <25 mg/L (aHR 2.77, CI 1.31–5.85, *P* = 0.008), and ≥10% vs. <10% weight loss prior to ART (aHR 2.31, CI 1.05–5.11, *P* = 0.038). A positive TB symptom score [36] was less predictive of IRIS than weight loss alone (one of the four components of the score) (aHR 2.23, CI 0.95–5.24, *P* = 0.066).

There were no statistically significant risk factors for Paradox-MC IRIS in multivariable analysis (Figure 4c). However, on univariate analyses, a baseline CD4^+^ count of ≤50 vs. >100 cells/µL was predictive (crude HR 1.90, CI 1.26–6.63, *P* = 0.012; aHR 1.97, CI 0.81–4.81, *P* = 0.14) and a CD4^+^ count of 51–100 cells/µL was of borderline significance (crude HR 2.25, CI 0.91–5.60, *P* = 0.081; aHR 1.60, CI 0.61–4.20, *P* = 0.34). When analysed as a continuous variable, each 50 cell decline in CD4^+^ count was associated with a 1.4-fold increased hazard (CI 1.06–1.84, *P* = 0.018). The nature, distribution and duration of pre-ART mucocutaneous conditions were not associated with subsequent paradoxical IRIS.

Risk factors for Unmask-MC IRIS on multivariable analysis (Figure 4d) were current TB (aHR 2.00, CI 1.02–3.93, *P* = 0.045) and previous TB (aHR 2.30, CI 1.21–4.37, *P* = 0.011), vs. those with no history of TB, mild anaemia of Hb 10–12 vs. >12 g/dL (aHR 1.98, CI 1.04–3.78, *P* = 0.037), but not Hb <10 g/dL, and use of co-trimoxazole for at least one month prior to ART vs. starting co-trimoxazole concurrently with ART (aHR 1.97, CI 1.06–3.65, *P* = 0.032). Of note, when possible and probable IRIS were combined, there was a similar magnitude of effect for the various risk factors for all IRIS subtypes, but the confidence intervals were wider.

## Discussion

This large cohort study of 498 patients initiating ART in Durban, South Africa provides the most comprehensive evidence to date on the frequency, clinical spectrum, timing, outcome, and programmatic impact of IRIS relative to other clinical events following ART initiation in a sub-Saharan African setting. Particular strengths of our study include the prospective data collection, rigorous ascertainment and assessment of all clinical events, a standardised approach to defining IRIS with inclusion of cases at the milder end of the spectrum of IRIS, and differentiation between paradoxical and unmasking IRIS events and between OIs and less serious conditions.

In this study, the cumulative incidence of IRIS was 22.9%, or 65 cases per 100 PY, of which 64% were unmasking and 36% paradoxical. The incidence rates of the different IRIS subtypes were: paradoxical major OI, 13.3% of patients with an existing OI at the time of ART initiation; unmasking major OI, 5.0% of the entire cohort; paradoxical mucocutaneous, 9.3% of patients with relevant pre-existing conditions; and unmasking mucocutaneous, 10.4% of the entire cohort. One quarter of all IRIS events were due to TB, with other OIs accounting for only 10 cases (7%). The remaining 68% of IRIS events were due to mucocutaneous conditions, mainly immune reconstitution folliculitis, a well-recognised but poorly quantified IRIS phenomenon [37], [38], [39], followed by viral skin conditions such as herpes simplex, warts and herpes zoster.

Our overall IRIS rate and clinical spectrum is comparable to a London cohort of predominantly black African patients [2], which reported 22.7% cumulative incidence (20% due to major OIs or HBV, and 80% to dermatological conditions), but higher than the 10–17% reported from studies in other RLS [1], [4], [5]. This may be partly explained by the low ascertainment of mucocutaneous IRIS events in these studies, lower rates of paradoxical TB-IRIS [1] and possible missed ascertainment of early IRIS events due to death or loss to follow-up (median IRIS onset 48 days compared to 15 days in our study). Our observed 4.8% incidence of unmasking TB-IRIS and combined 0.8% incidence of unmasking and paradoxical cryptococcal IRIS were similar to those reported in studies from other RLS for TB (3.7–5.9%) [1], [25], [40] and cryptococcal disease (0.3–1.6%) [19]. The incidence of mucocutaneous IRIS (19.1%) was more consistent with results from retrospective studies conducted in higher income settings (9–20% [2], [3], [6]) than in RLS (2–8% [1], [4], [5]).

The wide range of incidence of IRIS reported across studies may be explained by variations in patient-, site- and setting-specific factors [41], quality of screening prior to ART, or by case ascertainment and definition methods. Comparisons across studies are further constrained by the failure in most studies to discriminate between paradoxical and unmasking presentations, and the lack of a standardised case definition for IRIS. In the absence of clear immunopathological correlates or diagnostic assays, the differentiation of IRIS from other competing diagnoses and particularly unmasking IRIS from a new infection, or paradoxical IRIS from natural history of pre-existing disease, remains challenging. While no single case definition can comprehensively address all possible presentations of IRIS, the progressive development and use of both generic [10], [27], [42] and pathogen-specific [9], [19] case definitions in this and future studies of IRIS will improve reliability and comparability across studies.

We also evaluated the relative importance of IRIS compared to early non-IRIS clinical events. IRIS accounted for almost one quarter of all clinical events and deaths and one fifth of hospitalisations in the initial 24 weeks after ART initiation, which was similar to the contribution of non-IRIS new infections. Of the 114 patients who developed any type of IRIS event, 7% died, similar to the IRIS mortality of 5% and 9% reported in two studies from RLS [1], [5]. However, among the subgroup of patients with IRIS related to major OIs, there was a much higher morbidity and mortality (16.3% died, 34.9% were hospitalised, and 4.7% discontinued ART). Overall, there was no apparent negative impact of IRIS on ART adherence and retention, although follow-up was more intensive in our study than would be usual in most settings. Drug toxicity accounted for few events or hospitalisations, and no deaths, but almost half of ART discontinuations and regimen changes.

The baseline characteristics of our study population (predominantly young, female, with a high prevalence of WHO stage 3–4 disease and very low baseline CD4^+^ counts) are typical of other high HIV prevalence settings in sub-Saharan Africa [1], [5], [43], [44], [45], [46], [47], [48], [49]. We therefore extrapolated our findings to estimate the wider programmatic impact of IRIS. By 2009, approximately 972,000 patients had initiated ART in South Africa [50]. Assuming a 5% incidence of unmasking OI-IRIS, a 20% prevalence of co-infection with major OIs at ART initiation, a 13% incidence of paradoxical IRIS, and case fatality and hospitalisation rates of major OI-IRIS of 13.6% and 27.3%, we estimate that IRIS may have caused around 10,000 deaths and 20,000 hospital admissions in the South African ART programme alone since inception. If we apply the same assumptions to the 2.2 million individuals who have initiated ART across the ten countries with the largest ART programmes, IRIS may have accounted for as many as 23,000 deaths in these countries. The impact of IRIS is likely to increase as ART programmes in Africa are integrated with inpatient services, and ART is increasingly offered to those with major OIs and more advanced disease. However, it is important to emphasise that the effects of IRIS were similar to the number of events, deaths and hospitalisations caused by new non-IRIS infections, but slightly less than those caused by pre-existing conditions.

We identified some important differences in risk factor profiles between paradoxical and unmasking, and major OI-associated and mucocutaneous IRIS, consistent with these being distinct entities with differing pathogenesis, although these subgroup analyses were limited by small numbers. Consistent with previous studies, lower CD4^+^ count [1], [2], [3], [7], [16], [18], [26] and higher VL [3], [15], [30] at baseline were predictors of paradoxical OI (mainly TB)-IRIS, but not unmasking IRIS, together with shorter period of pre-ART duration of OI/TB therapy OI-IRIS [13], [16], [28], [29], [30], [31], [51], which is likely to be related to a higher residual pathogen load. In contrast with several published studies, the magnitude and rate of CD4^+^ immune reconstitution [6], [15], [32], [33] and VL reduction following ART initiation [13], [26], [28], [33] were not predictive of any type of IRIS. However, comparison across studies is difficult because of the potential confounding effect of a low baseline CD4^+^ count, and the use of CD4^+^ and VL responses in defining IRIS in some studies [6], [13], [15], [26], [28], [32].

For unmasking OI (predominantly TB) IRIS, the risk factors we identified were all consistent with the presence of sub-clinical disease, in particular lymphadenopathy present on chest X-ray, low haemoglobin, greater than 10% weight loss, and high baseline CRP. All were associated with at least a two- to three-fold increased risk of IRIS, and in the case of lymphadenopathy, a nine-fold risk. There are few existing data on risk factors for unmasking IRIS. One recent small study from Uganda of unmasking TB-IRIS identified both a low BMI and high CRP as independent predictors [25], but an Indian study identified no specific risk factors [51]. This is the first study to examine risk factors specific for mucocutaneous IRIS – the most common type of IRIS. Not surprisingly, with such a diverse range of conditions encompassing viral and bacterial aetiologies, identification of common risk factors was less productive. A low baseline CD4^+^ count was weakly predictive of paradoxical MC IRIS, and mild anaemia and a history of current or past TB were associated with unmasking MC IRIS.

There are several practical implications of our findings for reducing IRIS-associated morbidity and mortality in ART programmes. First, earlier HIV diagnosis and initiation of ART in accordance with revised WHO guidelines at a CD4^+^ count of 350 cells, and certainly before a decline to below 200 cells, remains the most important strategy to reduce the prevalence of major OIs, and the incidence of most serious and life-threatening IRIS and non-IRIS events. We estimate that for each 50 cell increase in baseline CD4 count at ART initiation, there would be a 17% reduction in the risk of any IRIS (95% CI 6–26%), and a 43% reduction in all-cause mortality (95% CI 19–60%).

Second, to reduce the likelihood of paradoxical OI IRIS, there is a need to optimise pre-ART OI management and timing of ART. Consistent with other reports, we found that a shorter duration of pre-ART OI/TB therapy was predictive of OI-IRIS [13], [16], [28], [29], [30], [31], [51], which is likely to be related to a higher residual pathogen load. The optimal timing of ART in patients with OIs requires balancing greater risk of IRIS after early initiation against continuing high mortality with delayed ART. Several RCTs of TB show a mortality benefit from earlier ART in TB [52], [53], [54], while one RCT suggested a benefit of deferral for ten weeks with cryptococcal infection [55]. While there are limitations in using observational data to inform this debate, our data can be viewed in conjunction with these RCTs and other IRIS cohort studies.

Third, there is a need to improve screening for OIs, particularly TB and cryptococcus, prior to ART initiation, to reduce the development of unmasking IRIS of major OIs, which are associated with a particularly poor outcome [46], [56], [57]. Several features suggestive of sub-clinical TB were predictive for unmasking TB in our study, suggesting that existing practices for pre-ART screening and treatment are inadequate. HIV programmes in high TB prevalence settings should incorporate a simple validated symptom score [36], [58] (with additional chest x-ray and sputum examination as required) into routine pre-ART evaluation, as recommended in WHO 2010 guidelines on intensified case finding [59]. This will be further enhanced by the increasing availability of rapid point of care tests for TB diagnosis [60]. Screening for cryptococcal antigen prior to ART initiation in patients with a CD4^+^ count less than 100 cells with pre-emptive fluconazole treatment in those who are serum CrAg positive has been shown to be a cost-effective intervention to reduce mortality [61], [62], [63] and this is now a recommendation for high cryptococcal prevalence settings in recent WHO advice [64]. Further operational research is needed to determine the feasibility and impact of a serum CrAg “screen and treat” strategy [65], [66].

More intensive monitoring of selected patients at high risk of IRIS (e.g. CD4^+^ count <50 cells, known OI, unexplained symptoms) in the initial few weeks after starting ART is a further strategy to reduce IRIS-related mortality by facilitating prompt management of severe IRIS. IRIS is generally self-limiting and interruption of ART is rarely indicated, but patients may require reassurance in the face of protracted symptoms to prevent discontinuation of or poor adherence to ART. Few national HIV guidelines from RLS contain guidance on recognition and management of IRIS. There is a need for both better health care worker training in this area as well as improved patient awareness.
